# Evaluation of Oxidative Stress Response Related Genetic Variants, Pro-oxidants, Antioxidants and Prostate Cancer

**DOI:** 10.3934/medsci.2015.4.271

**Published:** 2015-09-10

**Authors:** Nicole Lavender, David W. Hein, Guy Brock, La Creis R. Kidd

**Affiliations:** 1 Department of Pharmacology and Toxicology and James Graham Brown Cancer Center, University of Louisville, Louisville, KY; 2 Department of Bioinformatics and Biostatistics, University of Louisville, Louisville, KY

**Keywords:** Prostate cancer, oxidative stress, multifactor dimensionality reduction, gene-gene interactions, gene-environment interactions, genome wide association study, Cancer Genetics Markers of Susceptibility

## Abstract

**Background:**

Oxidative stress and detoxification mechanisms have been commonly studied in Prostate Cancer (PCa) due to their function in the detoxification of potentially damaging reactive oxygen species (ROS) and carcinogens. However, findings have been either inconsistent or inconclusive. These mixed findings may, in part, relate to failure to consider interactions among oxidative stress response related genetic variants along with pro- and antioxidant factors.

**Methods:**

We examined the effects of 33 genetic and 26 environmental oxidative stress and defense factors on PCa risk and disease aggressiveness among 2,286 men from the Cancer Genetic Markers of Susceptibility project (1,175 cases, 1,111 controls). Single and joint effects were analyzed using a comprehensive statistical approach involving logistic regression, multi-dimensionality reduction, and entropy graphs.

**Results:**

Inheritance of one *CYP2C8 rs7909236* T or two *SOD2 rs2758331* A alleles was linked to a 1.3- and 1.4-fold increase in risk of developing PCa, respectively (*p*-value = 0.006–0.013). Carriers of *CYP1B1 rs1800440GG, CYP2C8 rs1058932TC* and, *NAT2* (*rs1208GG, rs1390358CC, rs7832071TT*) genotypes were associated with a 1.3 to 2.2-fold increase in aggressive PCa [*p*-value = 0.04–0.001, FDR 0.088–0.939]. We observed a 23% reduction in aggressive disease linked to inheritance of one or more *NAT2 rs4646247* A alleles (*p* = 0.04, FDR = 0.405). Only three *NAT2* sequence variants remained significant after adjusting for multiple hypotheses testing, namely *NAT2 rs1208, rs1390358*, and *rs7832071*. Lastly, there were no significant gene-environment or gene-gene interactions associated with PCa outcomes.

**Conclusions:**

Variations in genes involved in oxidative stress and defense pathways may modify PCa. Our findings do not firmly support the role of oxidative stress genetic variants combined with lifestyle/environmental factors as modifiers of PCa and disease progression. However, additional multi-center studies poised to pool genetic and environmental data are needed to make strong conclusions.

## 1. Introduction

Oxidative stress is a condition in which the amount of reactive oxygen species (ROS) produced by pro-oxidants exceeds the amount removed by anti-oxidants [1,2]. ROS are highly reactive electrophiles that cause damage to biomolecules (i.e., DNA and proteins) when elevated [1,2]. This imbalance may lead: (1) to oxidized DNA bases, disrupted cell signaling, cellular transformations, altered protein structure, function as well as activation; (2) increased cellular proliferation; (3) decreased cell death; (4) accumulation of cellular damage; (5) and ultimately tumorigenesis [1,2]. Several cancers, including Prostate cancer (PCa) are linked to imbalances between pro-oxidation and anti-oxidation factors [3–5]. Men with PCa possess lower antioxidant enzyme levels in prostate tissues compared to both healthy controls and men with benign prostatic hyperplasia (BPH) [3]. Also, it has been demonstrated that PCa tissues contain higher amounts of ROS and oxidative DNA damage than normal prostate tissues [6]. In addition, *in vitro* studies have found ROS linked to PCa progression and more aggressive phenotypes (i.e., increased cell proliferation, anchorage-independent growth, and migration) [7,8].

Pro-oxidant factors include endogenous metabolic enzymes and exogenous exposures, including but not limited to meat- and cigarette-derived procarcinogens. A number of observation and/or cell or animal model assays have evaluated pro-oxidant exposures from cigarette smoking and pro-oxidant agents from cooked meats [e.g., heterocyclic amines (HCAs)] in relation to prostate cancer [9–17]. Although cigarette smoke may contribute to carcinogenesis based on its chemical composition, its role in PCa remains controversial. On one hand, a cohort study with over 22,000 men in the Physicians' Health Study (PHS) did not observe a significant association between smoking and overall PCa risk [18]. Conversely, a population-based case control study of 752 subjects demonstrated a 2.7-fold increase in PCa mortality risk among patients who self-reported as cigarette smokers at the time of diagnosis compared to non-smokers [12]. In addition, another report revealed current smokers had a 69% higher risk of PCa mortality compared to non-smokers [HR (95%CI) = 1.69 (1.25–2.27)] [19]. Meat-derived pro-oxidants including HCAs, such as 2-amino-1-methyl-6-phenylimidazo[4,5-*b*]pyridine (PhIP), 2-amino-3,8-dimethylimidazo[4,5-*b*]quinoxaline (MeIQx), and 2-amino-3,4,8-trimethylimidazo[4,5-*f*]quinoxaline (DiMeIQx), induce various cancers in rodents, including prostate cancer [15,20]. However, these pro-carcinogens must undergo metabolic activation to exert their genotoxic and carcinogenic effects by metabolic activation enzymes [9,15,20,21].

The body tries to protect itself from the carcinogenic effects of oxidative stress by maintaining homeostatic ROS levels. This entails the use of exogenous nutrients and endogenous metabolic/antioxidation enzymes (e.g., catalase, epoxide hydrolase, superoxide dismutase). Suppression of oxidative stress, presumably through a protective diet, retards cancer development and disease progression, including PCa [22–25]. For instance, intake of fruits and vegetables high in antioxidants (e.g., carotenoids, vitamins C & E, and selenium) protect cells from oxidative stress [22–24]. Compounds found in cruciferous vegetables (e.g., glucosinolates, isothiocyanates, flavonoids) protect cells from DNA damage, induce apoptosis, and inhibit cell proliferation of PCa [24]. Some flavonoids have antioxidant properties and bind to free radicals. Sequestration of ROS may ultimately decrease cancer development [24]. Vitamin E is a major lipid-soluble antioxidant in cell membranes with the capacity to scavenge free radicals, induce apoptosis, inhibit expression of Prostate Specific Antigen as well as Androgen Receptor mRNA, and reduce protein kinase C activity [23,25]. In addition, vitamin C is a potent ROS scavenger that can also induce apoptosis and reduce lipid peroxidation in cellular membranes [23,25]. Similar to Vitamin C, selenium has been shown to induce apoptosis, as well as inhibit cellular proliferation and angiogenesis [24].

Endogenous antioxidant enzymes are a major cellular oxidative stress defense mechanism in the removal of ROS [1,26]. These enzymes reduce ROS to less reactive species and thereby prevent cellular damage [1,26,27]. For example, superoxide dismutases (SODs) scavenge superoxide radicals and convert them to hydrogen peroxide molecules [26]. Reactive hydrogen peroxide is then subsequently removed by either catalase (CAT) or glutathione peroxidases (GPX1) [2,26,27]. Other antioxidative-related gene products important in detoxification and/or metabolism of ROS or pro-carcinogens include cytochrome P450s (CYPs), epoxide hydrolase (EPHX1), uridine 5'-diphospho-(UDP)-glucuronosyltransferases (UGTs), sulfotransferases (SULTs), *N*-acetyltransferases (NATs) and glutathione S-transferases (GSTs) [15,20,28,29]. Phase II metabolizing enzymes (UGTs, GSTs, SULTs) conjugate oxidized xenobiotics or ROS by transferring a glucuronic acid, glutathione, and sulfate group, leading to the production of less reactive, water soluble compounds that are readily excreted into the bile and urine [20,28]. To produce less reactive water-soluble compounds, UGT enzymes transfer a glucuronic acid, SULTs catalyze sulfate conjugation, and GSTs catalyze the conjugation of ROS to glutathione to produce less reactive water-soluble compounds [20,27]. Following oxidation by CYPs [15,20], EPHX1 converts epoxides from aromatic compounds to more water soluble dihydrodiols that can be excreted into the urine or bile [28]. NATs (i.e., NAT1 and NAT2) are phase II-metabolizing enzymes that catalyze detoxification of aromatic amines [30–32]. Hence, NAT1 and NAT2 are particularly important to the detoxification of carcinogens found in cigarette smoke.

Unfortunately, in some cases oxidative stress response related metabolic reactions can convert pro-oxidants derived from cigarette smoke or meat to more reactive intermediates [10]. For instance, when cigarette-derived PAHs, such as benzo[a]pyrene (B[a]P), undergo metabolic activation by cytochrome P450s this reaction leads to the generation of ROS, namely epoxides [33]. These highly reactive species can lead to oxidative DNA damage and possibly tumor formation, particularly by causing mutations in the tumor suppressor p53 gene [20]. Moreover, prior to exerting their genotoxic effects, meat-derived HCAs (e.g., PhIP, MeIQx, DiMeIQx) must undergo metabolic activation. CYPs catalyze the *N*-hydroxylation of HCAs, which undergo further metabolic activation by NATs or SULTs to form *N*^2^-acetoxylated or *N*^2^-sulfonyloxylated metabolites [34,35]. Similar to B[a]P, these highly reactive compounds can form DNA adducts that may lead to tumor formation, if left unrepaired [34,35]. Bioactivation to damaging reactive metabolites can also occur with endogenous ROS generated from cellular processes (e.g., respiration, electron-transport chain) [6,20,33]. Although, SODs scavenge superoxide radicals, this reaction produces hydrogen peroxide, which can lead to the formation of more reactive ROS if not eliminated [6,20,33]. Without its removal by CAT or GPX1, hydrogen peroxide can interfere with cellular signaling [6,20,33].

Although oxidative stress response related genetic variants, as well as pro- and antioxidants have been implicated in PCa etiology, the associations are not accepted across all observational studies [3,10,15,17,22,36–43]. The lack of consistent findings is partially due to failure to consider multiple genetic and environmental factors along with dietary antioxidants that may jointly modify PCa susceptibility and disease aggressiveness. To address this shortcoming, we examined the single and joint modifying effects of 33 oxidative stress response related genetic variants and 26 pro- and antioxidants in relation to prostate cancer using data available through the Cancer Genetic Markers of Susceptibility (CGEMS) and the National Cancer Institute (NCI) Prostate, Lung, Colon, and Ovarian (PLCO) Cancer Screening Trial databases [44–46]. Our analyses incorporated a comprehensive statistical strategy that included both traditional [i.e., logistic regression (LR)] and advanced [e.g., multifactor dimensionality reduction (MDR) and hierarchical interaction graphs] methodologies. These advanced tools not only allowed us to validate our LR models, but also provided a way to examine and visualize non-linear interactions. Furthermore, MDR has > 80% statistical power interactions to detect gene-gene and gene-environment interactions, even in the presence of small sample sizes (i.e., ≥ 200 cases, ≥ 200 controls). Studies such as this one are critical to enhancing our understanding of the role of oxidative stress in PCa development. Comprehensive analyses of genetic as well as environmental factors are needed in order to model complex interactions that contribute to this disease.

## 2. Materials and Methods

Our study population consists of nationally available genetic data from 2,286 men of European-descent (488 non-aggressive and 687 aggressive cases, 1,111 controls) collected through the PLCO Cancer Screening Trial [45–47]. This randomized, well-designed, multi-center trial was coordinated by the NCI [44]. Between 1993 and 2001, the PLCO Trial recruited men ages 55–74 years to evaluate the effect of screening on disease specific mortality, relative to standard care. All participants signed informed consent documents approved by both the NCI and University of Louisville institutional review boards. Access to clinical and background data collected through examinations and questionnaires was approved for use by the PLCO. Selected data for this population is summarized in Supplemental Tables A–D.

Several criteria were used for the selection of PLCO trial participants. Men were included in the current analysis if they had a baseline Prostate Specific Antigen (PSA) measurement before October 1, 2003, completed a baseline questionnaire, returned at least one Annual Study Update, and had available SNP profile data through the CGEMS data portal (http://cgems.cancer.gov/). For PCa screening, blood samples were collected and men received a PSA test and Digital Rectal Exam (DRE). Subsequent to the initial screen, participants received a PSA and DRE annually for three to five years, consecutively. Men who had PSA levels > 4 ng/mL or abnormal DRE were referred to their health care provider for follow-up care.

The PLCO Trial identified 1,175 PCa cases (488 non-aggressive and 687 aggressive). Incident cases were selected from various sources including: screening exams; reports from patients, physicians, or relatives; or linkage with the National Death Index or linkage with the state cancer registries. Incident PCa cases were pathologically confirmed with either aggressive (Gleason score 7 ≥ or tumor stage III/IV) or non-aggressive (Gleason score < 7 or tumor stage I/II) disease, based on Gleason Score and tumor stage at diagnosis. Since incident cases were defined as individuals diagnosed after the first year of follow-up, men receiving a diagnosis prior to one year of follow-up were excluded from the study.

### 2.1. Collection of dietary information and carcinogen exposure

Data for dietary and life style habits as well as supplement usage were collected from comprehensive questionnaires completed by study participants around the time of enrollment into the trial. For patient characteristics and lifestyle factors, risk categories were designated using guidelines recommended by the United States Department of Agriculture (USDA) Report of Dietary Guidelines and the NIH Office of Dietary Supplements [48,49]. More specifically, a subject was considered high risk if they: had a body mass index (BMI) greater than 30; consumed more than 3000 calories daily; ate less than 4 servings of fruits and 5 servings of vegetables, per day; participated in less than 30 minutes of physical activity each day; or consumed more than two alcoholic beverages daily [48,49]. Similarly, participants were considered high risk if they obtained less than the minimum daily recommended amount of Vitamins A, C, and E, Zinc, or Selenium. For variables related to meat consumption and cooking methods, as well as exposure to meat-derived carcinogens (i.e., MeIQx, DiMeIQx, PhIP, B[a]P) were divided into quartiles using data collected from the control subjects. The 1st quartile was used as the low risk category. These categories included daily total meat intake as well the amount of white (i.e., chicken and fish), processed, or red meats. Red meat consumption was also stratified by type or cooking duration into non-processed, rare/medium-well, and well-/very-well done. For meat-derived carcinogens, the minimal exposure group for each variable served as the low risk group.

### 2.2. Gene selection

A panel of 33 candidate genes was generated from genes involved in antioxidation and detoxification mechanisms based on published PCa epidemiology studies as well as pathway databases and tools, including KEGG, *Kyoto Encyclopedia of Genes and Genomes* (www.genome.jp/kegg), BioCarta (www.biocarta.com), ProteinLounge (www.proteinlounge.com), Ingenuity (www.ingenuity.com), and SNPs3D (www.SNPs3D.org) [50–55]. KEGG, BioCarta, and ProteinLounge were used to visualize protein-protein interactions essential to managing oxidative stress [50–54]. Ingenuity pathway analysis software was used to build a network of oxidative stress response related genes and interactive maps demonstrating important interactions based on published reports and/or other functional/pathway databases (e.g., KEGG and the Gene Ontology) [50–53]. These tools combined provide important molecular interactions and genes not readily found by literature search or other traditional methods.

A query of 33 candidate genes generated a SNP list of 209 variants in the CGEMS database. From these results, we selected sequence variants that were: (1) detected within an exon, 2.5 kb upstream of the gene, 2.5 kb downstream of intron 1, or 2.5 kb downstream of the gene; (2) had a minor allele frequency > 1% reported in the National Center for Biotechnology Information (NCBI) Entrez SNP, (www.ncbi.nlm.nih.gov); and (3) had an observed genotype frequency among controls that did not significantly deviate from the Hardy-Weinberg Equilibrium (HWE *p* < 0.005). This reduced our list of 209 SNPs in 33 genes to 33 SNPs detected in 19 pro- and antioxidative-related genes, which are listed in Supplemental Table E [28,56].

There was a minimal genotype failure rate (< 5%) for all 33 SNPs among disease-free men in the current study. The most commonly occurring genotype among controls was used to impute missing genotype data.

### 2.3. The impact of individual oxidative stress response related factors on prostate cancer

We evaluated 33 oxidative stress response related SNPs among 2,286 men of European descent (488 non-aggressive cases, 687 aggressive cases and 1,111 controls) in relation to PCa outcomes using LR analyses. To assess whether inheritance of at least one minor pro-/antioxidative allele modified the risk of developing PCa, we tested for significant differences in the distribution of homozygous major, heterozygous, or homozygous minor genotypes between cases and controls using the chi-square test of homogeneity. A case-case analysis was used to evaluate the relationship between oxidative stress-related alleles and aggressive PCa. For this analysis, we examined the distribution and inheritance of pro-/antioxidative genes comparing men with high tumor grade or stage (Gleason score ≥ 7, stage III/IV) to those with a lower grade or stage of disease (Gleason score < 7, stage I/II).

The associations between PCa outcomes and oxidative stress-related factors, expressed as odds ratios (ORs) and corresponding 95% confidence intervals (95% CIs), were estimated using unconditional multivariate LR models, adjusted for potential confounders (i.e., age and family history of PCa). LR analyses for PCa development were conducted using the major/common genotype or low risk lifestyle factor as the referent category. All chi-square test and LR analyses were conducted using SAS 9.2 (SAS Institute Inc, Cary, NC). Adjustments for multiple comparisons were made using False Discovery Rate (FDR). Models were considered significant if the FDR *p*-value ≤ 0.20.

### 2.4. Statistical power

We conducted calculations to determine the statistical power of our sample size to detect significant relationships between oxidative stress response-related sequence variants and PCa outcomes. The expected risk estimates of our study were estimated by specifying values for a number of parameters, including a minor allele frequency (MAF) of at least 20%, National Cancer Institute's estimate of PCa disease prevalence (19%), statistical power (80%), and pre-disposing variant = 1. For risk models (case versus control), the number of cases was 1,175 and controls were 1111. For the disease aggressiveness models (aggressive versus non aggressive), the number of cases was 687 (aggressive PCa cases) and the number of controls was 488 (non-aggressive cases). We assumed prostate cancer risk was in complete linkage disequilibrium with an oxidative stress response related predisposing variant (*r^2^* = 1.0). Based on our sample sizes, we have > 80% power to detect genetic markers with odds ratios (ORs) of ≥ 1.4 (or 0.71 for protective effects) for PCa risk and ≥ 1.5 (or 0.67 for protective effects) for aggressiveness. These estimates are based on the use of the additive genetic model with 1 degree of freedom (df). Calculations were performed using Power for Genetic Association Version 2 Software [58].

### 2.5. Analysis of gene-gene and gene-environment interactions using multi-factor dimensionality reduction (MDR)

We used MDR 2.0 (SourceForge, Inc, Sourceforge.net) to evaluate the single- and joint- modifying effects of genetic and environmental oxidative stress response related factors in relation to PCa and aggressive disease. The MDR software is open-source and freely available online [59]. This method is able to detect and characterize high-order interactions in case-control or case-only studies, and remain effective with relatively small sample sizes [60]. MDR has excellent statistical power (> 80%) to identify gene-gene or gene-environment interactions even in the presence of 5% genotyping error, 5% missing data, and/or in small sample sizes (i.e., ≤ 200 cases and controls) [60]. With MDR, multi-locus genotypes are pooled into high-risk and low-risk groups, reducing high-dimensional data to a single variable dimension and permitting an investigation of gene-gene or gene-environment interactions. This one-dimensional multi-locus genotype variable is evaluated for its ability to classify and predict a disease outcome through cross-validation and permutation testing. Finally, among all of the gene-gene combinations a single model is selected that maximizes the case-to-control ratio of the high-risk groups, while minimizing classification and prediction errors. MDR uses a 10-fold cross validation to estimate the testing accuracy of a model by leaving out one-tenth of the data as an independent test set. The model is developed on nine-tenths of the data and then evaluated on the remaining test set. This process is repeated for each one-tenth of the data, and the resulting prediction accuracies are averaged. The prediction accuracy is calculated as the average of prediction accuracies across each of the 10 cross-validation subsets [61,62]. The model with the greatest Cross Validation Consistency (CVC) [e.g. ≥ 8/10] and highest prediction accuracies [e.g., Average Testing Accuracy (ATA)] is selected as the best predictor of disease outcome [61,62]. MDR models are validated by comparing the average CVC to the distribution of the average consistencies under the null hypothesis of no association, derived empirically from 1,000 permutations. The null hypothesis is rejected when the upper-tail Monte Carlo *p*-value is ≤ 0.05. The version of MDR used in this project allows for the incorporation and adjustment of multiple covariates [63]. To remove the covariate effect, we integrated two sampling methods (i.e., over- and under-sampling). This approach is computationally efficient and allows for the adjustment of multiple covariates without significantly increasing computational burden. Inclusion of covariates allows estimates of specificity, sensitivity, and overall predictive accuracy with and without the genetic or environmental factors in order to assess the gains in predictive ability afforded by the putative risk factors.

In the current study, significant interaction models identified by MDR were further assessed by LR modeling to calculate interaction terms using a significance cut-off level of 0.05.

### 2.6. Visualization of interaction models using interaction entropy algorithms, hierarchical graphs and statistical epistasis network

Interaction entropy algorithm, based on information theory, is a method to verify, visualize, and interpret combination effects identified by MDR [60,64–66]. Orange software was used to perform interaction entropy analyses among selected genetic and environmental factors in relation to PCa risk and disease progression. Interaction entropy uses information gain (IG) to gauge whether interactions between two or more factors provide more information about PCa outcomes relative to each factor considered independently [60,64–66]. Individual as well as all possible pairwise loci are assigned an IG percentage score in relation to disease risk or aggressiveness (scores < 5% are typical) [60,64–66]. Pairwise SNP combinations were deemed important if the pairwise IG was greater than the IG for each individual locus [(IG_SNP_1+ SNP_2_ > IG_SNP_1_) and (IG_SNP_1+ SNP_2_ > IG_SNP_2_)] [60,65–67].

## 3. Results

CGEMS and PLCO study participants consisted of middle-aged non-Hispanic men of European descent, ranging in ages between 55 and 81. Compared to controls, PCa cases were more likely to have a family history of prostate cancer (11.4% versus 6.3%) and PSA levels ≥ 4 ng/mL (48.5% versus 6.5%), as depicted in Supplemental Table A. There were no marked differences in body mass index (BMI) and lifestyle characteristics (i.e., physical activity, daily dietary or vitamin/mineral intakes, alcohol consumption), comparing cases to controls or aggressive and non-aggressive cases, as shown in Supplemental Tables A–D. However, there were more current smokers among the controls (*p* = 0.022) and more never smokers among the cases versus controls (*p* = 0.045).

### 3.1. Impact of individual oxidative stress response related sequence variants on prostate cancer outcomes

Out of 33 oxidative stress-related sequence variants obtained from the CGEMS database, we identified two targets that were individually associated with PCa risk. Inheritance of one minor *CYP2C8 rs7909236* T allele was linked to a 1.3-fold increase in PCa risk [OR (95%CI) = 1.27 (1.07–1.51); *p* = 0.006, *p*-trend = 0.033, FDR = 0.649], as summarized in Table 1. Additionally, inheritance of the *SOD2 rs2758331* AA genotype was associated with a 1.4-fold increase in PCa risk [OR (95%CI) = 1.36 (1.08–1.70); *p* = 0.013, *p*-trend = 0.016, FDR = 0.538], as shown in Table 1.

In relation to disease aggressiveness, we found six SNPs associated with aggressive PCa, as shown in Table 2. Inheritance of two minor *CYP1B1 rs1800440* G, *CYP2C8 rs1058932* T, *NAT2 rs1208* G, *NAT2 rs1390358* C, or *NAT2 rs7832071* T allele was associated with a 1.3 to 2.2-fold increase in disease aggressiveness (*p*-values = 0.001–0.04, FDR = 0.088–0.939) relative to those with the referent genotype. Conversely, there was a 23% reduction in aggressive PCa among men who possessed at least one minor *NAT2 rs4646247* A allele when compared to those with the reference genotype [OR (95%CI) = 0.77 (0.60–0.98); *p* = 0.044, FDR = 0.405]. Among the aforementioned PCa disease aggressiveness risk alleles, only *NAT2 rs1208, NAT2 rs1390358* and *NAT2 rs7832071* remained statistically significant after adjusting for FDR (*p*-value = 0.088–0.158).

### 3.2. Combination effects of oxidative stress response related factors on prostate cancer outcomes

Upon examination of the joint effects our genetic and environmental panel on PCa risk using MDR, we detected a significant interaction between *CYP2C8 rs7909236* and *GSTP1 rs1695*. These SNPs were selected as the best two factor model for predicting disease risk [CVC = 10/10; ATA = 0.545; *p* = 0.013], as depicted in Table 3. However, this finding was not confirmed by LR analysis (*p*-value for interaction = 0.100; *p*-trend = 0.016), as shown in Supplemental Table F. However, the entropy graph revealed that this interaction was mainly driven by *CYP2C8*, as depicted in Supplemental Figure 1. More specifically, *CYP2C8* alone had an IG value of 0.31%, while *CYP2C8* and *GSTP1* yield an IG of 0.31%. Hence, there is no additional information gained comparing the two-factor model (i.e., *CYP2C8-GSTP1*) to *CYP2C8 rs7909236* alone or *GSTP1 rs1695* alone. There were no significant gene-environment or gene-gene interaction MDR models selected as effective predictors of PCa risk.

With regards to disease aggressiveness, MDR did not show any significant gene-gene or gene-environment interaction models linked to disease aggressiveness (*p* ≥ 0.375), as depicted in Table 4. Even though a complex interaction among daily intake of white, processed and well-done red meat was selected as the best three factor MDR model, in relation to aggressive disease, the low cross validation consistency score (CVC < 8) preempted further consideration.

## 4. Discussion

Oxidative stress occurs when there is an increase in the production or decrease in the removal of ROS [1,2,33,68]. Endogenous and exogenous ROS sources can contribute to oxidative stress [1,2,33,68]. This includes products generated from normal cellular respiration and metabolic processes as well as exposure to environmental carcinogens including, PAHs and HCAs [1,33]. Excessive oxidative stress can produce DNA base changes, damage tumor suppressors, enhance proto-oncogene expression, and induce malignant transformation of cells [1,2,33,68]. The damaging effects of ROS may be further exacerbated by susceptibilities in antioxidation/detoxification genes and compromise the capacity to manage oxidative stress. Increased exposure to environmental ROS sources can exacerbate this effect. Consequently, oxidative stress response related gene variants associated with decreased ROS capacity, combined with elevated ROS levels due to environmental factors may increase the risk of PCa development. To evaluate this hypothesis we assessed the effects of 33 pro-/antioxidative-related sequence variants along with 26 environmental oxidative stress response related factors in relation to PCa risk and disease aggressiveness. This analysis was performed using a comprehensive statistical approach that included traditional (i.e., LR) as well as advanced methodologies (i.e., MDR and entropy graphs). Data related to dietary habits, vitamin/ supplement intake, and exposure to meat- and cigarette-derived carcinogens was collected from 2,286 CGEMS project participants (687 aggressive and 488 non-aggressive cases, 1111 controls).

Among the 33 sequence variants examined in the current study, three NAT2 loci were predictive of aggressive PCa among participants of the CGEMs GWAS study. Commensurate with our study findings, *NAT2* (rs1208, rs1390358, rs7832071) were significantly related to PCa (*p*-value = 0.001). These markers remained significant after adjusting for multiple hypotheses testing (FDR *p* value ≤ 0.158). NAT2 enzyme activity can either detoxify or bioactivate many xenobiotics and these effects are largely substrate dependent [69]. *NAT2 rs1208* has a substitution of G for A at position 803, which causes a lysine to arginine amino acid change at position 268 [69]. This variant is associated with the rapid acetylation phenotype similar to the referent NAT2*4 allele [69,70]. Previous studies have confirmed this variant does not alter mRNA or protein expression and activity [69,70]. However, this *NAT2* rs1208 SNP exists with several slow *NAT2* haplotypes (i.e., *5F, *5G, *6C) [71–75]. Unfortunately, the CGEMS project does not have genotype data available for these other variants within the aforementioned NAT2*5/*6 haplotypes. Therefore, we cannot eliminate the possibility that other *NAT2* alleles may contribute to the positive association we observed between rs1208 and PCa and disease aggressiveness. To our knowledge, there are no published data or functional predictions regarding the other intronic *NAT2* SNPs (i.e., rs1390358 and rs7832071). These two intronic SNPs may influence miRNA splicing or miRNA binding sites, resulting in alterations in mRNA and/or protein levels [56]. Therefore, the increased risk of developing aggressive PCa among carriers of the *NAT2* (rs1390358 and rs7832071) variant alleles may be linked to decreased detoxification or increased bioactivation of pro-oxidants.

The role of oxidative stress response related factors in relation to PCa outcomes has undergone evaluation within a few observational studies. However, reported findings are inconsistent. Koutros and colleagues evaluated gene-environment interactions among nearly 120 polymorphisms across multiple metabolizing genes (*CYP1A1, CYP1A2, CYP1B1, GSTA1, GSTM1, GSTM3, GSTP1, NAT1, NAT2, SULT1A1, SULT1A2*, and *UGT1A* locus) and meat-derived HCAs in relation to PCa susceptibility within a subset of participants selected from the PLCO Trial [15]. Meat-derived carcinogen exposures were estimated using questionnaire data regarding meat consumption and cooking method for a study population of 1126 cases (473 non-aggressive, 654 aggressive) and 1127 controls [15]. From this analysis, possession of at least one or more variant *GSTM3 rs11102001* was associated with increased PCa risk among subjects in the highest percentile of DiMeIQx intake compared to subjects in the lowest percentile [OR (95%CI) = 2.3 (1.2–4.7). HCA-SNP analyses revealed a significant interaction among *GSTM3 rs11102001*, MeIQx, and DiMeIQx (*p* = 0.001). This relationship remained significant after adjusting for multiple hypothesis testing (false discovery rate (FDR) = 0.20) [15]. Additional data from this same study suggests joint risk effects may exist among *GSTP1^105^Val* or the *UGT1A* locus; however, this interaction did not survive after adjusting for multiple comparisons (FDR > 0.03) [15]. Sharma and co-workers (2010) examined eight *NAT1* and seven *NAT2* polymorphic alleles, along with well-done red meat consumption in relation to PCa risk using a multi-ethnic cohort population (2106 cases, 2063 controls) [76]. Individual and multivariate statistical analyses were conducted using possession of NAT1*10 or ‘slow’ *NAT2* phenotypes and frequent consumption of well-done red meat designated as the high risk groups [76]. No single or combined risk effects were observed between variant *NAT1* or *NAT2* acetylators and well-done red meat intake in relation to PCa [76].

Unlike previous reports that examined the role of pro-/antioxidative targets in PCa susceptibility, our study utilized a sophisticated statistical approach to evaluate single and joint modifying effects of genetic as well as environmental factors in relation to PCa and aggressive disease. MDR and entropy graphs allowed us to model gene-gene as well as gene-environment interactions within a large panel of factors and study population. Furthermore, we were able to evaluate several markers that have not been investigated in previous publications using SNP data collected through the CGEMS project. Consistent with previously published reports, we were not able to detect significant gene-environment and gene-gene interactions associated with PCa risk or disease aggressiveness [15,76–78]. Our inability to detect significant joint modifying effects was partially attributed to the lack of commonly studied or functional genetic variants within the CGEMS database. For instance, it may be worthwhile to analyze SNPs in genes such as, glutathione peroxidases, peroxiredoxins, and thioredoxins. Future studies can address this concern by utilizing targeted sequencing strategies to secure additional markers relevant in metabolic activation, antioxidation, and detoxification pathways. Also, actual exposure levels from cigarette- and meat-derived carcinogens instead of questionnaire estimates may permit more significant gene-environment interactions. The addition of more oxidative stress related genetic variants and more accurate exposures will strengthen epidemiological studies and help elucidate the role of oxidative stress mechanisms in prostate carcinogenesis.

## Supplementary Material

01

## Figures and Tables

**Table 1:** Association of selected antioxidative SNPs on prostate cancer risk.

Marker (Alleles and position)	Allele	Cases N (%)	Controls N (%)	OR (95%CI)	Adj OR (95%CI)*	*p*-value	*p*-trend	FDR
*CYP2C8*	GG	626 (54.2)	659 (59.6)	1.00 (reference)	1.00 (reference)	0.024	0.038	0.649
rs7909236	TG	468 (40.5)	386 (35.0)	1.27 (1.07–1.51)	1.27 (1.07–1.51)	0.006		
G96819420T	TT	61 (5.3)	60 (5.4)	1.07 (0.74–1.55)	1.05 (0.72–1.53)	0.730		
	TG+TT	529 (45.8)	446 (40.4)	1.21 (0.96–1.53)	1.24 (1.05–1.47)	0.112		
*SOD2*	CC	292 (25.1)	316 (28.4)	1.00 (reference)	1.00 (reference)	0.051	0.016	0.538
rs2758331	AC	574 (49.3)	555 (49.9)	1.12 (0.92–1.37)	1.13 (0.92–1.37)	0.250		
C160025060A	AA	298 (25.6)	241 (21.7)	1.34 (1.06–1.69)	1.36 (1.08–1.72)	0.013		
	AC+AA	872 (74.9)	796 (71.6)	1.19 (0.98–1.43)	1.19 (0.99–1.44)	0.072		

* adjusted for age and family history.

**Table 2:** Association of selected antioxidative SNPs with aggressive prostate cancer.

Marker (Alleles and position)	Allele	Cases N (%)	Controls N (%)	OR (95%CI)	Adj OR (95%CI)*	p-value	p-trend	FDR
*CYP1B1*	AA	774 (66.5)	766 (68.8)	1.00 (reference)	1.00 (reference)	0.089	0.388	0.939
rs1800440	AG	350 (30.1)	309 (27.8)	0.95 (0.73–1.22)	0.94 (0.73–1.22)	0.667		
A38209790G	GG	40 (3.4)	38 (3.4)	2.14 (1.03–4.44)	2.15 (1.04–4.46)	0.041		
	AG+GG	390 (33.5)	347 (31.2)	1.02 (0.80–1.31)	1.02 (0.80–1.30)	0.861		
*CYP2C8*	CC	446 (65.7)	341 (71.5)	1.00 (reference)	1.00 (reference)	0.088	0.033	0.276
rs1058932	TC	208 (30.6)	122 (25.6)	1.32 (1.01–1.72)	1.31 (1.01–1.71)	0.039		
C96786851T	TT	25 (3.7)	14 (2.9)	1.38 (0.71–2.70)	1.37 (0.70–2.68)	0.344		
	TC+TT	233 (34.3)	136 (28.5)	1.33 (1.03–1.71)	1.30 (1.01–1.68)	0.028		
*NAT2*	AA	221 (32.1)	169 (34.6)	1.00 (reference)	1.00 (reference)	0.001	0.007	0.119
rs1208	AG	304 (44.2)	247 (50.6)	0.94 (0.72–1.22)	0.94 (0.72–1.22)	0.649		
A18302596G	GG	163 (23.7)	72 (14.7)	1.73 (1.23–2.44)	1.75 (1.24–2.46)	0.002		
	AG+GG	467 (67.9)	319 (65.3)	1.12 (0.87–1.43)	1.12 (0.88–1.44)	0.377		
*NAT2*	TT	230 (33.6)	173 (35.8)	1.00 (reference)	1.00 (reference)	0.000	0.008	0.088
rs1390358	TC	310 (45.2)	251 (52.0)	0.94 (0.73–1.22)	0.94 (0.73–1.22)	0.657		
T18297035C	CC	145 (21.2)	59 (12.2)	1.88 (1.31–2.69)	1.88 (1.31–2.70)	0.001		
	TC+CC	455 (66.4)	310 (64.2)	1.09 (0.86–1.39)	1.10 (0.86–1.41)	0.483		
*NAT2*	GG	367 (53.6)	227 (47.0)	1.00 (reference)	1.00 (reference)	0.114	0.059	0.405
rs4646247	AG	263 (38.4)	212 (43.9)	0.78 (0.61–0.99)	0.77 (0.60–0.98)	0.044		
G18303188A	AA	55 (8.0)	44 (9.1)	0.78 (0.51–1.20)	0.77 (0.50–1.18)	0.266		
	AG+AA	318 (46.4)	256 (53.0)	0.78 (0.62–0.98)	0.76 (0.60–0.96)	0.037		
*NAT2*	CC	222 (32.3)	172 (35.2)	1.00 (reference)	1.00 (reference)	0.001	0.005	0.158
rs7832071	TC	307 (44.6)	247 (50.6)	0.96 (0.74–1.25)	0.96 (0.74–1.25)	0.776		
C18301560T	TT	159 (23.1)	69 (14.1)	1.78 (1.26–2.52)	1.80 (1.27–2.55)	0.001		
	TC+TT	466 (67.7)	316 (64.7)	1.14 (0.89–1.46)	1.15 (0.90–1.46)	0.286		

* adjusted for age and family history.

**Table 3:** Multi-Dimensionality reduction models for antioxidative-related polymorphisms and prostate cancer risk.

Best Model	Cross Validation Consistency (CVC)*	Average Testing Accuracy*	Permutation Testing *p*-value*

One Factor	10/10	0.526	0.080
*CYP2C8_rs7909236*			
Two Factor			
*CYP2C8_rs7909236*	10/10	0.545	0.013
*GSTP1_rs1695*			
Three Factor			
*CYP2C8_rs7909236*	3/10	0.502	0.403
*GSTP1_rs1695*			
*NAT1_rs4921581*			
Four Factor			
*GSTM2_rs638820*			
*GSTM3_rs7483*	5/10	0.536	0.021
*GSTP1_rs6591256*			
*NAT2_rs1112005*			

* Adjusted for age and family history of prostate cancer.

**Table 4:** Multi-Dimensionality reduction models for antioxidative-related targets and prostate cancer aggressiveness.

Best Model	Cross Validation Consistency (CVC)*	Average Testing Accuracy*	Permutation Testing p-value*

One Factor	8/10	0.510	0.440
*CYP2C8_rs7909236*			
Two Factor			
*CYP2C8_rs7909236*	3/10	0.504	0.375
*DiMeIQx*			
Three Factor			
*White_meat_intake*	7/10	0.534	0.035
*Processed_meat*			
*Well_done_red_Meat*			
Four Factor			
*White_meat_intake*			
*Processed_meat*	5/10	0.525	0.117
*Rare_red_Meat*			
*Well_done_red_meat*			

* Adjusted for age and family history of prostate cancer.

## List of Abbreviations

PCa: prostate cancer
ROS: reactive oxygen species
SOD: superoxide dismutases
CAT: catalase
GPX: glutathione peroxidase
CYP: cytochrome P450s
EPHX1: epoxide hydrolase
UGT: uridine 5'-diphospho-(UDP)-glucuronosyltransferase
SULT: sulfotransferase
NAT: *N*-acetyltransferase
GST: glutathione S-transferase
PhIP: 2-amino-1-methyl-6-phenylimidazo[4,5-*b*]pyridine
MeIQx: 2-amino-3,8-dimethylimidazo[4,5-*b*]quinoxaline
DiMeIQx: 2-amino-3,4,8-trimethylimidazo[4,5-*f*]quinoxaline
SNP: single nucleotide polymorphism
CGEMS: Cancer Genetic Markers of Susceptibility
PLCO: Prostate, Lung, Colon, and Ovarian
PSA: Prostate Specific Antigen
DRE: Digital Rectal Exam
LR: logistic regression
OR: odds ratio
MAF: minor allele frequency
MDR: multifactor dimensionality reduction
CVC: cross validation consistency
ATA: average testing accuracy
IG: information gain

## References

[1] Sies H (1997). Oxidative stress: oxidants and antioxidants. Exp Physiol.

[2] Halliwell B (2007). Oxidative stress and cancer: have we moved forward?. Biochem J.

[3] Choi JY, Neuhouser ML, Barnett M (2007). Polymorphisms in oxidative stress-related genes are not associated with prostate cancer risk in heavy smokers. Cancer Epidemiol Biomarkers Prev.

[4] Waris G, Ahsan H (2006). Reactive oxygen species: role in the development of cancer and various chronic conditions. J Carcinog.

[5] Miyake H, Hara I, Kamidono S (2004). Oxidative DNA damage in patients with prostate cancer and its response to treatment. J Urol.

[6] Khandrika L, Kumar B, Koul S (2009). Oxidative stress in prostate cancer. Cancer Lett.

[7] Kumar B, Koul S, Khandrika L (2008). Oxidative stress is inherent in prostate cancer cells and is required for aggressive phenotype. Cancer Res.

[8] Pathak SK, Sharma RA, Steward WP (2005). Oxidative stress and cyclooxygenase activity in prostate carcinogenesis: targets for chemopreventive strategies. Eur J Cancer.

[9] Caceres DD, Iturrieta J, Acevedo C (2005). Relationship among metabolizing genes, smoking and alcohol used as modifier factors on prostate cancer risk: exploring some gene-gene and gene-environment interactions. Eur J Epidemiol.

[10] Cross AJ, Peters U, Kirsh VA (2005). A prospective study of meat and meat mutagens and prostate cancer risk. Cancer Res.

[11] Fleshner NE, Klotz LH (1998). Diet, androgens, oxidative stress and prostate cancer susceptibility. Cancer Metastasis Rev.

[12] Gong Z, Agalliu I, Lin DW (2008). Cigarette smoking and prostate cancer-specific mortality following diagnosis in middle-aged men. Cancer Causes Control.

[13] Huncharek M, Haddock KS, Reid R (2010). Smoking as a risk factor for prostate cancer: a meta-analysis of 24 prospective cohort studies. Am J Public Health.

[14] Kolonel LN (2001). Fat, meat, and prostate cancer. Epidemiol Rev.

[15] Koutros S, Berndt SI, Sinha R (2009). Xenobiotic metabolizing gene variants, dietary heterocyclic amine intake, and risk of prostate cancer. Cancer Res.

[16] Rohrmann S, Genkinger JM, Burke A (2007). Smoking and risk of fatal prostate cancer in a prospective U.S. study. Urology.

[17] Sinha R, Park Y, Graubard BI (2009). Meat and meat-related compounds and risk of prostate cancer in a large prospective cohort study in the United States. Am J Epidemiol.

[18] Lotufo PA, Lee IM, Ajani UA (2000). Cigarette smoking and risk of prostate cancer in the physicians' health study (United States). Int J Cancer.

[19] Watters JL, Park Y, Hollenbeck A (2009). Cigarette smoking and prostate cancer in a prospective US cohort study. Cancer Epidemiol Biomarkers Prev.

[20] Boelsterli UA (2007). Mechanistic Toxicology: the molecular basis of how chemicals disrupt biological targets.

[21] Cross AJ, Sinha R (2004). Meat-related mutagens/carcinogens in the etiology of colorectal cancer. Environ Mol Mutagen.

[22] Kushi LH, Byers T, Doyle C (2006). American Cancer Society Guidelines on Nutrition and Physical Activity for cancer prevention: reducing the risk of cancer with healthy food choices and physical activity. CA Cancer J Clin.

[23] Chan R, Lok K, Woo J (2009). Prostate cancer and vegetable consumption. Mol Nutr Food Res.

[24] Ma RW, Chapman K (2009). A systematic review of the effect of diet in prostate cancer prevention and treatment. J Hum Nutr Diet.

[25] Kovacic P, Jacintho JD (2001). Mechanisms of carcinogenesis: focus on oxidative stress and electron transfer. Curr Med Chem.

[26] Mates JM, Perez-Gomez C, Nunez dCI (1999). Antioxidant enzymes and human diseases. Clin Biochem.

[27] Aydin A, rsova-Sarafinovska Z, Sayal A (2006). Oxidative stress and antioxidant status in non-metastatic prostate cancer and benign prostatic hyperplasia. Clin Biochem.

[28] (2011). National Center for Biotechnology Information (NCBI) website.

[29] Gamage N, Barnett A, Hempel N (2006). Human Sulfotransferases and Their Role in Chemical Metabolism. Toxicolog Sci.

[30] Hein DW (2002). Molecular genetics and function of NAT1 and NAT2: role in aromatic amine metabolism and carcinogenesis. Mutat Res.

[31] Gross GA, Turesky RJ, Fay LB (1993). Heterocyclic aromatic amine formation in grilled bacon, beef and fish and in grill scrapings. Carcino Genesis.

[32] Badawi AF, Hirvonen A, Bell DA (1995). Role of aromatic amine acetyltransferases, NAT1 and NAT2, in carcinogen-DNA adduct formation in the human urinary bladder. Cancer Res.

[33] Sikka SC (2003). Role of oxidative stress response elements and antioxidants in prostate cancer pathobiology and chemoprevention--a mechanistic approach. Curr Med Chem.

[34] Zhou SF, Wang B, Yang LP (2010). Structure, function, regulation and polymorphism and the clinical significance of human cytochrome P450 1A2. Drug Metab Rev.

[35] Metry KJ, Neale JR, Doll MA (2010). Effect of rapid human N-acetyltransferase 2 haplotype on DNA damage and mutagenesis induced by 2-amino-3-methylimidazo-[4,5-f]quinoline (IQ) and 2-amino-3,8-dimethylimidazo-[4,5-f]quinoxaline (MeIQx). Mutat Res.

[36] Autrup JL, Thomassen LH, Olsen JH (1999). Glutathione S-transferases as risk factors in prostate cancer. Eur J Cancer Prev.

[37] Beer TM, Evans AJ, Hough KM (2002). Polymorphisms of GSTP1 and related genes and prostate cancer risk. Prostate Cancer Prostatic Dis.

[38] Gsur A, Haidinger G, Hinteregger S (2001). Polymorphisms of glutathione-S-transferase genes (GSTP1, GSTM1 and GSTT1) and prostate-cancer risk. Int J Cancer.

[39] Wadelius M, Autrup JL, Stubbins MJ (1999). Polymorphisms in NAT2, CYP2D6, CYP2C19 and GSTP1 and their association with prostate cancer. Pharmaco genetics.

[40] Forsberg L, de FU, Morgenstern R (2001). Oxidative stress, human genetic variation, and disease. Arch Biochem Biophys.

[41] Kang D, Lee KM, Park SK (2007). Functional variant of manganese superoxide dismutase (SOD2 V16A) polymorphism is associated with prostate cancer risk in the prostate, lung, colorectal, and ovarian cancer study. Cancer Epidemiol Biomarkers Prev.

[42] U.S. Human and Health Services (2005). 11th Report on Carcinogens.

[43] Zheng W, Lee SA (2009). Well-done meat intake, heterocyclic amine exposure, and cancer risk. Nutr Cancer.

[44] Cancer Genetic Markers of Susceptibility (CGEMS) (2008).

[45] Gohagan JK, Prorok PC, Hayes RB (2000). The Prostate, Lung, Colorectal and Ovarian (PLCO) Cancer Screening Trial of the National Cancer Institute: history, organization, and status. Control Clin Trials.

[46] Hayes RB, Sigurdson A, Moore L (2005). Methods for etiologic and early marker investigations in the PLCO trial. Mutat Res.

[47] Hasson MA, Fagerstrom RM, Kahane DC (2000). Design and evolution of the data management systems in the Prostate, Lung, Colorectal and Ovarian (PLCO) Cancer Screening Trial. Control Clin Trials.

[48] US Department of Health and Human Services and US Department of Agriculture (2005). Dietary Guidelines for Americans.

[49] National Institutes of Health Office of Dietary Supplements.

[50] Kanehisa M, Araki M, Goto S (2008). KEGG for linking genomes to life and the environment. Nucleic Acids Res.

[51] Kanehisa M, Goto S, Hattori M (2006). From genomics to chemical genomics: new developments in KEGG. Nucleic Acids Res.

[52] Kanehisa M, Goto S (2000). KEGG: kyoto encyclopedia of genes and genomes. Nucleic Acids Res.

[53] Ingenuity Systems (2010). Ingenuity Pathways Analysis.

[54] BioCarta LLC (2009). BioCarta.com. http://BioCarta.com.

[55] Yue P, Melamud E, Moult J (2006). SNPs3D: candidate gene and SNP selection for association studies. BMC Bioinform.

[56] Xu Z, Taylor JA (2009). SNPinfo: integrating GWAS and candidate gene information into functional SNP selection for genetic association studies. Nucleic Acids Res.

[57] Ramensky V, Bork P, Sunyaev S (2002). Human non-synonymous SNPs: server and survey. Nucleic Acids Res.

[58] Menashe I, Rosenberg PS, Chen BE (2008). PGA: power calculator for case-control genetic association analyses. BMC Genet.

[59] Moore JH, Gilbert JC, Tsai CT (2006). A flexible computational framework for detecting, characterizing, and interpreting statistical patterns of epistasis in genetic studies of human disease susceptibility. J Theor Biol.

[60] Andrew AS, Nelson HH, Kelsey KT (2005). Concordance of multiple analytical approaches demonstrates a complex relationship between DNA repair gene SNPs, smoking, and bladder cancer susceptibility. Carcino Genesis.

[61] Hahn LW, Ritchie MD, Moore JH (2003). Multifactor dimensionality reduction software for detecting gene-gene and gene-environment interactions. Bioinformatics.

[62] Moore JH (2004). Computational analysis of gene-gene interactions using multifactor dimensionality reduction. Expert Rev Mol Diagn.

[63] Gui J, Andrew AS, Andrews P (2010). A Robust Multifactor Dimensionality Reduction Method for Detecting Gene-Gene Interactions with Application to the Genetic Analysis of Bladder Cancer Susceptibility. Ann Hum Genet.

[64] Ritchie MD, Hahn LW, Moore JH (2003). Power of multifactor dimensionality reduction for detecting gene-gene interactions in the presence of genotyping error, missing data, phenocopy, and genetic heterogeneity. Genet Epidemiol.

[65] Jakulin A, Bratko I, Lavrac N, Gamberger D, Blockeel H, Todorovski L (2003). Analyzing attribute depedencies. PKDD 2003.

[66] Jakulin A, Bratko I, Smrike D (2003). Attribute interactions in medical data analysis.

[67] Demosar J, Zupan B (2004). Orange: From Experimental Machine Learning to Interactive Data Mining, White Paper.

[68] Coates PJ, Lorimore SA, Wright EG (2005). Cell and tissue responses to genotoxic stress. J Pathol.

[69] Hein DW, Fretland AJ, Doll MA (2006). Effects of single nucleotide polymorphisms in human N-acetyltransferase 2 on metabolic activation (O-acetylation) of heterocyclic amine carcinogens. Int J Cancer.

[70] Zang Y, Doll MA, Zhao S (2007). Functional characterization of single-nucleotide polymorphisms and haplotypes of human N-acetyltransferase 2. Carcino Genesis.

[71] Agundez JA, Olivera M, Ladero JM (1996). Increased risk for hepatocellular carcinoma in NAT2-slow acetylators and CYP2D6-rapid metabolizers. Pharmacogenetics.

[72] Agundez JA, Olivera M, Martinez C (1996). Identification and prevalence study of 17 allelic variants of the human NAT2 gene in a white population. Pharmacogenetics.

[73] Anitha A, Banerjee M (2003). Arylamine N-acetyltransferase 2 polymorphism in the ethnic populations of South India. Int J Mol Med.

[74] Patin E, Barreiro LB, Sabeti PC (2006). Deciphering the ancient and complex evolutionary history of human arylamine N-acetyltransferase genes. Am J Hum Genet.

[75] Woolhouse NM, Qureshi MM, Bayoumi RA (1997). A new mutation C759T in the polymorphic N-acetyltransferase (NAT2) gene. Pharmacogenetics.

[76] Sharma S, Cao X, Wilkens LR (2010). Well-done meat consumption, NAT1 and NAT2 acetylator genotypes and prostate cancer risk: the multiethnic cohort study. Cancer Epidemiol Biomarkers Prev.

[77] Nock NL, Tang D, Rundle A (2007). Associations between smoking, polymorphisms in polycyclic aromatic hydrocarbon (PAH) metabolism and conjugation genes and PAH-DNA adducts in prostate tumors differ by race. Cancer Epidemiol Biomarkers Prev.

[78] Koutros S, Andreotti G, Berndt SI (2011). Xenobiotic-metabolizing gene variants, pesticide use, the risk of prostate cancer. Pharmacogenet Genomics.

