# Supplementary material for: Evaluation of Oxidative Stress Response Related Genetic Variants, Pro-oxidants, Antioxidants and Prostate Cancer
Source: AIMS Med Sci. Author manuscript; Available in PMC 2015 Dec 10. (PMC4664461; doi:10.3934/medsci.2015.4.271)
Supplement: 01 [file NIHMS724455-supplement-01.pdf]

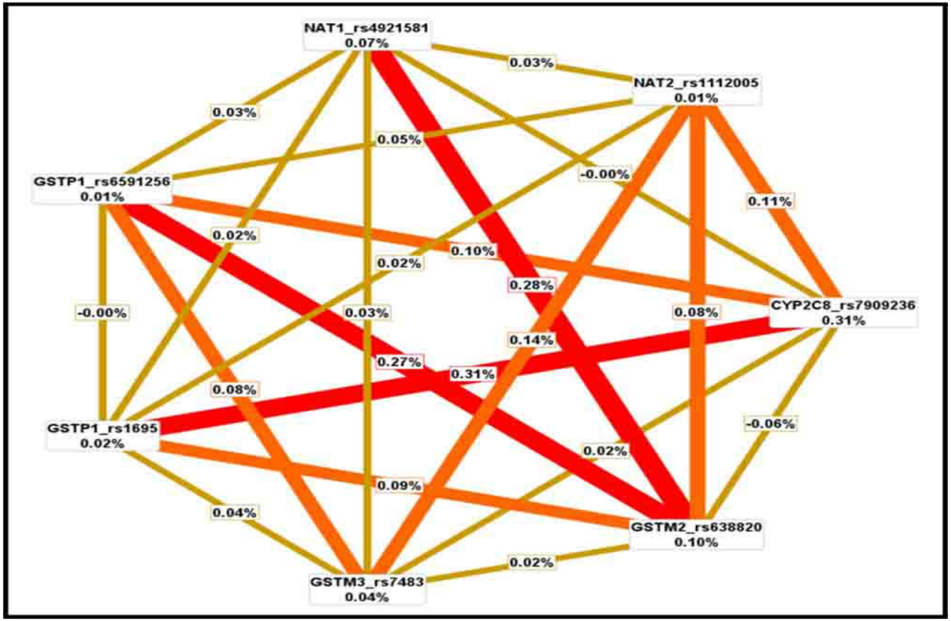

Supplemental Figure 1.

Supplemental Table

Supplemental Table A. Baseline characteristics by disease status among male participants of the CGEMS study.

Characteristics	Cases	Controls	*p* value[a]
Number of Participants, n	1,175	1,111	---
Age at diagnosis (yrs), median (range)	67 (55–81)	67 (55–80)	0.299
Age at enrollment (yrs), median (range)	65 (55–74)	64 (55–74)	0.094
Family History of Prostate Cancer, n (%)			
Yes	133 (11.4)	70 (6.3)	< 0.0001
No	1031 (88.7)	1041 (93.7)	
PSA (ng/ml),[b] n (%)			
< 4	569 (48.9)	1022 (93.5)	< 0.0001
≥ 4	564 (48.5)	71 (6.5)	
Missing	22 (1.9)	18 (1.6)	
DRE results,[b] n (%)			
Normal	398 (34.2)	537 (48.3)	< 0.0001
Abnormal, suspicious	472 (40.6)	438 (39.4)	
Abnormal, non-suspicious	234 (20.1)	75 (6.8)	
Missing	59 (5.1)	61 (5.5)	
**Lifestyle**			
Body Mass Index (BMI),[c] n (%)			
Underweight or normal	305 (26.2)	271 (24.4)	0.244

Overweight	612 (52.6)	574 (51.7)	0.648
Obese	246 (21.2)	266 (23.9)	0.111
Missing	0 (0.0)	0 (0.0)	
Kcal from diet (g/day),[c] n (%)			
2000–3000	559 (47.6)	522 (47.0)	0.821
< 2000	395 (33.6)	391 (35.2)	0.538
> 3000	209 (17.8)	198 (17.8)	0.926
Missing	12 (1.0)	0 (0.0)	
Fat from diet (g/day), median (IQR)			
Fat	73.1 (95.5–56.4)	72.7 (99.2–55.7)	0.884
Saturated	25.0 (32.4–18.6)	24.6 (34.0–18.5)	0.790
Missing	1 (0.4)	2 (0.9)	
Physically Active (at least 30 min/day),[c] n (%)	556 (47.3)	494 (44.5)	0.177
Currently	559 (47.6)	620 (55.8)	0.224
Since age 40	1 (0.1)	2 (0.2)	
Missing			
Tobacco Use, n (%)			
Never	477 (40.6)	421 (37.9)	0.045
Former	593 (50.5)	570 (51.3)	0.880
Current	93 (7.9)	120 (10.8)	0.022
Ever (Former & Current)	686 (58.4)	690 (62.1)	0.128
Alcohol Consumption (drinks/day),[b] n (%)			
≤ 2	960 (81.7)	923 (83.1)	0.736
> 2	203 (17.3)	188 (16.9)	
Missing	12 (1.0)	0 (0.0)	

Abbreviations: PSA, prostate specific antigen; DRE, digital rectal examination; [a]Differences in frequencies were tested by a Chi-square test of heterogeneity; Differences in continuous variables between cases and controls were tested using the Wilcoxon sum Rank test; [b]PSA given between year 0–5 & DRE given between year 0-3 of PLCO study; [c]Risk categories are based on values established in the 2005 USDA dietary guidelines & NIH office of dietary supplements.

Supplemental Table B. Dietary characteristics by disease status among male participants of the CGEMS study.

	Cases	Controls	*p* value[a]
Meat Consumption (g/day), median (IQR)			
Total meat	173.9 (118.4–254.2)	174.5 (129.3–252.9)	0.166
White Meat (chicken & fish)	42.6 (25.1–71.9)	44.6 (26.2–71.8)	0.478
Processed meat	11.4 (6.1–21.0)	11.4 (6.1–21.0)	0.646
Red Meat group	80.9 (47.6–125.0)	82.7 (53.9–124.2)	0.278
Red meat not processed	62.1 (38.6–95.7)	62.1 (38.6–95.7)	0.396
Red meat rare/med done	15.0 (3.8–33.6)	16.0 (3.9–32.4)	0.567
Red meat well/very well done	9.3 (4.1–19.8)	9.8 (4.9–19.8)	0.141
Meat-derived carcinogen exposure, median (IQR)			
MeIQx (ng/day)	22.3 (10.8–44.6)	23.9 (13.1–46.6)	0.009
DiMeIQx (ng/day)	1.0 (0.3–2.4)	1.2 (0.4–2.6)	0.016
PhIP (ng/day)	73.6 (32.9–141.7)	74.0 (36.7–156.8)	0.266
B[a]P (ng/day)	8.4 (1.4–42.2)	9.1 (1.7–44.6)	0.084
Fruit (servings/day),[b] n (%)			
≥ 4	975 (83.0)	952 (85.7)	0.219
< 4	188 (16.0)	159 (14.3)	
Missing	12 (1.0)	0 (0.0)	
Vegetables (servings/day),[b] n (%)			
≥ 5	907 (77.2)	831 (74.8)	0.073
< 5	256 (21.8)	280 (25.2)	
Missing	12 (1.0)	0 (0.0)	
Vitamin A (μg/day),[b] n (%)			
≥ 900	1054 (89.7)	1008 (90.6)	0.990
< 900	109 (9.3)	103 (9.3)	
Missing	12 (1.0)	0 (0.0)	

Vitamin C (mg/day),[b] n (%)			
≥ 75	1103 (93.9)	1042 (93.8)	0.245
< 75	61 (5.2)	71 (6.4)	
Missing	11 (0.9)	0 (0.0)	
Vitamin E (IU/day),[b] n (%)			
≥ 15	1014 (86.3)	952 (85.7)	0.273
< 15	150 (12.8)	161 (14.5)	
Missing	11 (0.9)	0 (0.0)	
Zinc (mg/day),[b] n (%)			
≥ 11	837 (71.2)	775 (69.8)	0.215
< 11	325 (27.7)	336 (30.2)	
Missing	13 (1.1)	0 (0.0)	
Selenium (μg/day),[b] n (%)			
≥ 55	1128 (96.0)	1085 (97.7)	0.324
< 55	34 (2.9)	26 (2.3)	
Missing	13 (1.1)	0 (0.0)	

Abbreviations: IQR, Interquartile Range; [a]Differences in frequencies were tested by a Chi-square test of heterogeneity; Differences in continuous variables between cases and controls were tested using the Wilcoxon sum Rank test; [b]Risk categories are based on values established in the 2005 USDA dietary guidelines & NIH office of dietary supplements.

Supplemental Table C. Baseline disease & lifestyle characteristics for PCa patients.

Characteristics	Aggressive Cases	Non-Aggressive Cases	*p* value[a]
Number of Participants, n	687	488	---
Age at diagnosis (yrs), Median (range)	67 (55–81)	66 (55–78)	0.083
Age at enrollment (yrs), Median (range)	64 (55–74)	65 (55–74)	0.080
Family History of Prostate Cancer, n (%)			
Yes	605 (88.1)	435 (89.1)	0.525
No	83 (12.1)	53 (10.9)	
PSA (ng/ml),[b] n (%)			
< 4	347 (50.5)	230 (48.0)	0.173
≥ 4	319 (46.4)	249 (52.0)	
Missing	21 (3.0)	9 (1.8)	
Gleason Score,[b] n (%)			
4	4 (0.6)	45 (9.8)	< 0.0001
5	18 (1.4)	133 (29.0)	
6	86 (12.5)	271 (59.2)	
7	459 (66.8)	8 (1.8)	
8	68 (9.9)	1 (0.2)	
9	44 (6.4)	0 (0.0)	
10	3 (0.4)	0 (0.0)	
DRE results,[b] n (%)			
Normal	241 (35.1)	159 (34.4)	0.435
Abnormal, suspicious	282 (41.0)	197 (42.6)	
Abnormal, non-suspicious	130 (18.9)	106 (23.0)	
Missing	34 (4.9)	26 (5.3)	
**Lifestyle**			
Body Mass Index (BMI),[c] n (%)			
Underweight or normal	180 (26.2)	127 (26.0)	0.126
Overweight	350 (50.9)	272 (55.8)	0.105
Obese	157 (22.9)	89 (18.2)	0.055
Missing	0 (0.0)	0 (0.0)	
Kcal from diet (g/day),[c] n (%)			
2000–3000	336 (48.9)	230 (47.1)	0.352
< 2000	237 (34.5)	161 (33.0)	0.591
> 3000	114 (16.6)	97 (19.9)	0.149
Missing	1 (0.9)	0 (0.0)	
Fat from diet (g/day), median (IQR)			
Fat	73.1 (57.7–94.6)	73.1 (56.4–98.2)	0.196
Saturated	25.0 (19.2–32.5)	25.0 (18.9–34.2)	0.114
Missing	1 (0.9)	0 (0.0)	

Physically Active (at least 30 min/day),[c] n (%)			
Currently	333 (48.5)	229 (46.9)	0.601
Since age 40	354 (51.5)	254 (52.1)	0.508
Missing	0 (0.0)	5 (1.0)	
Tobacco Use, n (%)			
Never	296 (43.1)	186 (38.1)	0.169
Former	335 (48.8)	265 (54.3)	0.061
Current	56 (8.2)	37 (7.6)	0.722
Ever (Former & Current)	391 (56.9)	302 (61.9)	0.088
Alcohol Consumption (drinks/day),[b] n (%)			
≤ 2	579 (84.3)	390 (79.9)	0.053
> 2	108 (15.7)	98 (20.1)	
Missing	0 (0.0)	0 (0.0)	

Abbreviations: PSA, prostate specific antigen; DRE, digital rectal examination; [a]Differences in frequencies were tested by a Chi-square test of heterogeneity; Differences in continuous variables between cases and controls were tested using the Wilcoxon sum Rank test; [b]PSA given between year 0– & DRE given between year 0–3 of PLCO study, Gleason Score represents best Gleason Score taken at prostatectomy or biopsy; [c]Risk categories are based on values established in the 2005 USDA dietary guidelines & NIH office of dietary supplements.

Supplemental Table D. Dietary characteristics by disease aggressiveness among male participants of the CGEMS study.

	Aggressive Cases	Non-Aggressive Cases	*p* value[a]
Meat Consumption (g/day), median (IQR)			
Total meat	174.5 (124.3–240.7)	174.5 (118.1–245.6)	0.918
White Meat (chicken & fish)	44.6 (25.6–66.6)	44.6 (27.6–71.4)	0.129
Processed meat	11.4 (5.9–21.4)	11.4 (5.2–23.5)	0.928
Red Meat group	82.7 (50.8–122.8)	82.7 (50.2–119.8)	0.992
Red meat not processed	62.1 (36.2–93.6)	62.1 (36.7–91.9)	0.831
Red meat rare/med done	16.0 (3.9–32.7)	16.0 (3.8–31.6)	0.838
Red meat well/very well done	9.8 (4.7–19.4)	9.8 (4.2–16.9)	0.496
Meat-derived carcinogen exposure, median (IQR)			
MeIQx (ng/day)	23.6 (11.9–44.6)	22.0 (11.0–39.0)	0.155
DiMeIQx (ng/day)	1.2 (0.4–2.3)	1.0 (0.3–2.1)	0.462
PhIP (ng/day)	74.0 (35.4–125.2)	77.0 (38.0–142.5)	0.081
B[a]P (ng/day)	9.1 (1.7–37.4)	9.1 (1.5–42.3)	0.968
Fruit (servings/day),[b] n (%)			
≥ 4	584 (85.0)	403 (82.6)	0.264
< 4	103 (15.0)	85 (17.4)	
Missing	0 (0.0)	0 (0.0)	
Vegetables (servings/day),[b] n (%)			
≥ 5	547 (79.6)	370 (75.8)	0.121
< 5	140 (20.4)	118 (24.2)	
Missing	0 (0.0)	0 (0.0)	
Vitamin A (μg/day),[b] n (%)			
≥ 900	619 (90.1)	444 (91.0)	0.636
< 900	68 (9.9)	44 (9.0)	
Missing	0 (0.0)	0 (0.0)	
Vitamin C (mg/day),[b] n (%)			
≥ 75	40 (3.4)	22 (4.5)	0.324
< 75	647 (94.2)	466 (95.5)	
Missing	0 (0.0)	0 (0.0)	
Vitamin E (IU/day),[b] n (%)			
≥ 15	93 (13.5)	59 (12.1)	0.472
< 15	594 (13.5)	429 (87.9)	
Missing	0 (0.0)	0 (0.0)	
Zinc (mg/day),[b] n (%)			
≥ 11	491 (71.5)	351 (71.9)	0.876
< 11	196 (28.5)	137 (28.1)	
Missing	0 (0.0)	0 (0.0)	

Selenium (μg/day),[b] n (%)			
≥ 55	24 (3.5)	11 (2.3)	0.218
< 55	663 (96.5)	477 (97.7)	
Missing	0 (0.0)	0 (0.0)	

Abbreviations: IQR, Interquartile Range; [a]Differences in frequencies were tested by a Chi-square test of heterogeneity; Differences in continuous variables between cases and controls were tested using the Wilcoxon sum Rank test; [b]Risk categories are based on values established in the 2005 USDA dietary guidelines & NIH office of dietary supplements.

Supplemental Table E. Selected antioxidative-related polymorphisms.

dbSNP ID[†]	Gene	Chr	Chr Position	Location	Nucleotide Change	MAF (%)	Amino Acid Change	Predicted Functional Consequence [54]
rs1001179	*CAT*	11	34416807	5'near gene (−206)	G > A	3.2–9.1		TFBS
rs564250	*CAT*	11	34415437	5'near gene (−1616)	C >T	1.7–4.5		TFBS
rs2470893	*CYP1A1*	15	72806502	3'near gene (+1540)	G > A	2.0–7.1		TFBS
rs1800440	*CYP1B1*	2	38209790	Exon 2	A > G	3.2–5.0	Asn453Ser	Possibly Damaging
rs11673270	*CYP2B6*	19	46212684	Intron 1	A > C	7.1		
rs2860840	*CYP2C18*	10	96485222	3'UTR (mRNA 1830)	C > T	11.6–13.3		miRNA
rs10509681	*CYP2C8*	10	96788739	Exon 8	T > C	1.7–4.2	Lys399Arg	Benign
rs1058932	*CYP2C8*	10	96786851	3'UTR (mRNA 1592)	C > T	2.7		mRNA
rs7909236	*CYP2C8*	10	96819420	5'near gene (−120)	G > T	3.3–4.4		TFBS
rs2480258	*CYP2E1*	10	135240981	Intron 1	G > A	3.5–4.2		
rs2515642	*CYP2E1*	10	135240894	Intron 1	T >C	3.5–4.3		
rs6413420	*CYP2E1*	10	135229710	5'near gene (−38)	G > T	3.4–4.2		Splicing; possibly damaging
rs1051740	*EPHX1*	1	222326368	Exon 4	T > C	10–12.5	Tyr113His	
rs1051741	*EPHX1*	1	222338964	Exon 2	C > T	1.8	Asn357Asn	Splicing; benign
rs2234922	*EPHX1*	1	222333141	Exon 5	A > G	5.3–7.1	His139Arg	
rs6917325	*GSTA1*	6	52774232	Intron 1	C > T	15.3–16.8		TFBS
rs563464	*GSTA3*	6	52883831	5'near gene (−1387)	C > T	4.2		TFBS
rs638820	*GSTM2*	1	109921948	5'near gene (−765)	C > T	25.9		Benign
rs7483	*GSTM3*	1	109991743	Exon 7	G > A	6.5–18.2	Val224Ile	Benign
rs1695	*GSTP1*	11	67109265	Exon 5	A > G	9.7–21.7	Ile105Val	
rs6591256	*GSTP1*	11	67106475	5'near gene (−1197)	A > G	16.7–25.0		miRNA
rs10888150	*NAT1*	8	18110406	5'near gene(−1489)	C > T	18.6–20		
rs4921581	*NAT1*	8	18115375	Intron	G > A	6.7–9.7		
rs7003890	*NAT1*	8	18121590	Intron 1	T > C	23.3–29.2		
rs7017402	*NAT1*	8	18112354	Intron 1	G > A	9.2–10.6		TFBS
rs8190870	*NAT1*	8	18125552	3'near gene (+452)	C > T	2.7–3.3		
rs1112005	*NAT2*	8	18300156	Intron 1	C > T	11.5–20		
rs1208	*NAT2*	8	18302596	Exon 2	A > G	16.1–20.6	Lys268Arg	Benign
rs1390358	*NAT2*	8	18297035	Intron 1	T > C	13.6–16.7		
rs4646247	*NAT2*	8	18303188	3'near gene (+224)	G > A	8.8–13.6		
rs7832071	*NAT2*	8	18301560	Intron 1	C > T	15.3–16.7		
rs2758331	*SOD2*	6	160025060	Intron 1	C > A	20–21.7		
rs6717546	*UGT1A1*	2	234464119	3'near gene (+174)	G >A	4.5–16.7		TFBS

Abbreviations: Chr, chromosome; UTR, untranslated region; TFBS, transcription factor binding site; miRNA, microRNA.

Supplemental Table F. Interaction models for antioxidative-related targets and prostate cancer risk.

Model	Minor Allele/ Group	# Minor Alleles/ Group	OR (95%CI)	Adj OR (95%CI)*	*p* value	Interaction *p* value	*p* trend
CYP2C8_rs7909236	T	0–1	1.00 (reference)	1.00 (reference)			
GSTP1_rs1695	G	2	1.30 (1.08–1.58)	1.30 (1.07–1.58)	0.007	0.100	0.016
		≥ 3	0.92 (0.67–1.26)	0.91 (0.66–1.26)	0.578		

*adjusted for age and family history.
